# Complement Opsonization of HIV-1 Enhances the Uptake by Dendritic Cells and Involves the Endocytic Lectin and Integrin Receptor Families

**DOI:** 10.1371/journal.pone.0023542

**Published:** 2011-08-11

**Authors:** Veronica Tjomsland, Rada Ellegård, Karlhans Che, Jorma Hinkula, Jeffrey D. Lifson, Marie Larsson

**Affiliations:** 1 Molecular Virology, Department of Clinical and Experimental Medicine, Linköping University, Linköping, Sweden; 2 AIDS and Cancer Virus Program, SAIC Frederick, Inc., National Cancer Institute at Frederick, Frederick, Maryland, United States of America; The University of Hong Kong, Hong Kong

## Abstract

Interaction with the complement system is an underappreciated aspect of HIV-1 infection; even in primary infection, complement fragments are found on virions with potential to affect the interplay between the virus and dendritic cells (DC). Since opsonization may affect the efficiency of uptake and the type of receptors utilized, we compared the interactions of DC with free HIV-1 (F-HIV) and complement opsonized HIV-1 (C-HIV). We demonstrate that C-HIV significantly enhanced the uptake by immature DC (IDC) and mature DC (MDC) and that the internalization rate was dependent on both opsonization of the virus and DC maturation state. Increased DC uptake of C-HIV was not due to opsonization related increased binding of virus to the surface of DC but rather increased internalization of C-HIV despite utilizing a similar repertoire of receptors as F-HIV. Both F-HIV and C-HIV interacted with C-type lectins, integrins, and CD4 and blocking these receptor families prevented HIV-1 from binding to DC at 4°C. Blocking integrins significantly reduced the binding and uptake of F-HIV and C-HIV implicating the involvement of several integrins such as β1-integrin, CR3, LFA-1, and α4β7. Distinctive for C-HIV was usage of β1-integrin and for F-HIV, usage of β7-integrin, whereas both F-HIV and C-HIV utilized both integrin chains of CR3. We have in this study identified the receptor types used by both F-HIV and C-HIV to bind to DC. Noteworthy, C-HIV was internalized more efficiently by DC than F-HIV, probably via receptor mediated endocytosis, which may entail different intracellular processing of the virus leading to both elevated infection and altered activation of HIV specific immune responses.

## Introduction

The complement system plays an active role in both the innate and adaptive immune system and is stringently controlled to not damage the host. While complement opsonization of pathogens often has direct antimicrobial functions and augments the immune response, complement also targets apoptotic cells and leads to their removal without triggering autoimmunity or inflammation [1]. HIV-1 is able to activate the complement cascade in body fluids, both in the absence and in the presence of virus specific antibodies [2]. Virions incorporate host cell derived proteins into their envelopes, including membrane cofactor protein (CD46), decay accelerating factor (CD55), and protectin (CD59), when budding from infected cells. These receptors can prevent the complement cascade from destroying the virus [3], and result in virions opsonized in inactivated complement fragment 3b (iC3b) and iC3d [3]. After the onset of HIV-1 infection, virions can exist as free, complement opsonized, or cell bound viruses [4] and following seroconversion, virions might be covered in both HIV-1 specific antibodies and complement fragments. The virions involved in mucosal transmission are expected to be opsonized by complement and other factors present in cervical secretions or seminal fluids. Therefore it is possible that interactions with target cells, including mucosal DC and T cells, involve different forms of opsonized HIV-1, both during transmission and through the course of infection.

Different subsets of DC are present as immature DC (IDC) in tissues such as skin and mucosa, where they survey their surroundings for danger signals, and as mature DC (MDC) in the lymphoid organs, where they are essential for the priming of T cell responses [5], [6]. DC express an array of receptors including the surface proteins CD4 and CCR5/CXCR4, which are the main receptors utilized by HIV-1 for infection of immune cells. In addition, they express several other receptors capable of binding and internalizing HIV-1, e.g. C-type lectins such as DC-SIGN, macrophage mannose receptor (MMR), Langerin, and syndecan-3, Fcγ-receptors, and complement receptors (CR) [5]. Of note, the expression levels of these receptors are dependent on the subtype and maturation status of the DC [6].

Complement opsonization of HIV-1 has been shown to enhance infectivity in vitro in the context of direct infection of monocytes, and IDC [2], [7], [8], [9], [10], [11]. A previous study showed that complement opsonized virions were more efficiently transferred from IDC to CD4+ T cells and that the transfer occurred in a CR3 and DC-SIGN dependent manner [9]. However, the role of DC-SIGN in DC trans-infection of T cells has recently been questioned [12], [13] and may depend on how the virions have been prepared and type of DC utilized. Pruenster et al found that complement opsonized HIV-1 (C-HIV) interacted with IDC in a DC-SIGN independent manner [11]. Furthermore, opsonization with complement and antibodies has been reported to both enhance and decrease the ability of HIV-1 to infect IDC [2], [10].

In this study, we examined binding and internalization of C-HIV and F-HIV by IDC and MDC and established the receptors involved. Uptake by IDC and MDC of HIV-1 coated with complement or complement in combination with HIV-specific antibodies was significantly enhanced compared to F-HIV. Furthermore, the level of bound and internalized virus was higher and the internalization of virions into trypsin insensitive compartments was faster in MDC compared to IDC. HIV-1 interacted with integrins, C-type lectins, and the CD4 receptor and simultaneous blocking of these receptors almost abolished virus binding to both IDC and MDC at 4°C. However, the exact receptors within the integrin and C-type lectin families utilized depended on whether the virions were coated with complement or free. DC-SIGN was involved in the binding of F-HIV to IDC, whereas this was not the case for C-HIV. C-HIV used β1- and β2-integrins, whereas F-HIV used β2- and β7-integrins for attachment to IDC. Of note, both F-HIV and C-HIV utilized both chains of CR3 (CD11b/CD18). The levels of C-HIV and F-HIV that bound to the surface of DC were comparable, whereas the level of internalized virus was significantly higher for C-HIV, suggesting that that complement opsonization allowed for more efficient receptor mediated uptake of virions as been shown for other pathogens.

## Results

### Complement opsonization of HIV-1 increased binding and endocytosis of virions by IDC and MDC, with IDC showing slower endocytic kinetics for HIV-1 than MDC

**Figure 1:**
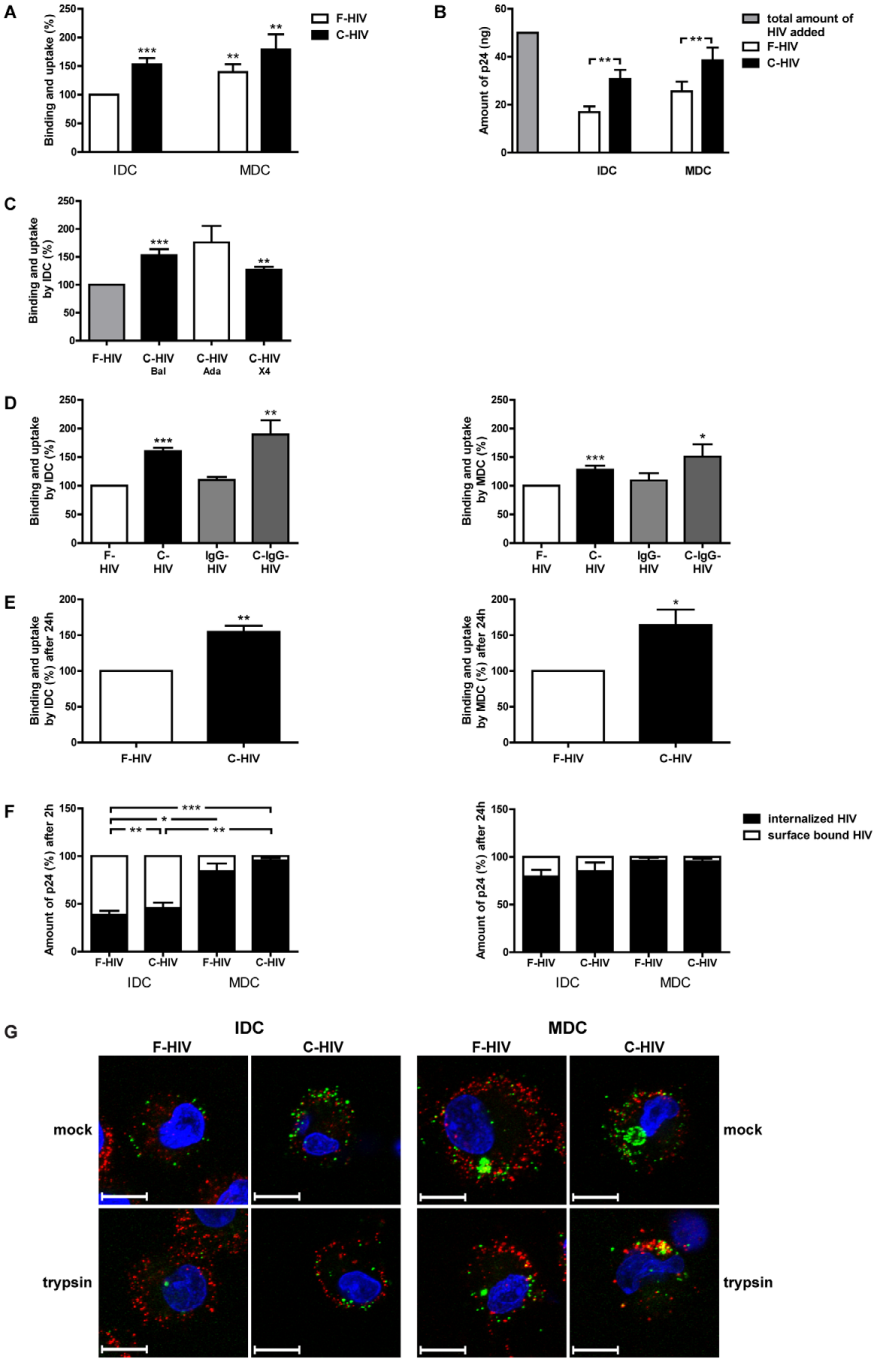
Complement opsonization of HIV-1 increased the binding and uptake by IDC and MDC. (**A–B**) 0.15×10^6^ IDC and MDC were incubated with 50 ng p24 free HIV-1_BaL_(F-HIV) or complement opsonized HIV-1_BaL_ (C-HIV) and the level of binding and uptake of HIV-1 after 2 h was measured by p24 ELISA (N = 22) and shown normalized against F-HIV (**A**) or as amount of p24 (**B**). (**C**) IDC were incubated with HIV-1_BaL_, HIV-1_ADA_, or HIV-1_MN_ either as F-HIV or C-HIV and the level of binding and uptake of HIV-1 after 2 h was measured by p24 ELISA (N = 3–4). (**D**) IDC and MDC were incubated with F-HIV, C-HIV, IgG opsonized (IgG-HIV), or complement IgG opsonized HIV-1_BaL_ (C-IgG-HIV) and the level of binding and uptake of HIV-1 after 2 h was measured by p24 ELISA (N = 8–60). (**E**) IDC and MDC were incubated with free or complement opsonized HIV-1_BaL_ and the level of binding and uptake of HIV-1 after 24 h was measured by p24 ELISA (N = 4–5). (**F**) IDC and MDC were incubated with F-HIV or C-HIV for 2 h or 24 h followed by mock or trypsin treatment of the IDC and MDC and the p24 levels were measured by ELISA (N = 46). (**G**) IDC and MDC were incubated with free or complement opsonized GFP-HIV-1_BaL_ (green) for 2 h followed by mock or trypsin treatment and stained with mAb targeting the CD1a receptor expressed by the DC (red) and DAPI (nucleus: blue) (N = 3). The binding and uptake of GFP-HIV-1_BaL_ was assessed by scanning confocal microscopy. Results were tested for statistical significance using a two-sided paired t-test and p<0.05 was considered statistically significant. Values have been normalized and free HIV has been set to 100%. All values± SEM. N = 3–50. * = p<0.05, ** = p<0.005, *** = p<0.0005.

We studied the capacity of HIV-1 to interact with (bind) and to be internalized (uptake) into immature DC (IDC) and mature DC (MDC), utilizing HIV-1_BaL_ (CCR5 tropic) either as free virions (F-HIV) or virions opsonized with complement from fresh human serum (C-HIV), HIV-1 specific antibodies (IgG-HIV), or both complement and antibodies (C-IgG-HIV). MDC bound/endocytosed significantly higher levels of both F-HIV (39%, p<0.005) and C-HIV (18% p<0.005) compared to IDC (**Fig. 1A**). The efficiency of the complement opsonization using fresh human serum was tested by heat inactivation as inactivating sera at 56°C for 30 minutes abolishes complement activation [9]. We found no increase in binding/uptake by IDC when using HIV-1 opsonized by heat inactivated sera, the levels were the same as for F-HIV (**Fig. S1**). Of the total virus that was added to the cells, IDC bound ∼35% of F-HIV and 62% of C-HIV and MDC bound ∼50% of F-HIV and 77% of C-HIV) (**Fig. 1B**), indicating that our system was saturated. To ensure the enhanced binding and uptake was not a unique feature for HIV-1_BaL_ we examined the effect complement opsonization had on the CCR5 tropic HIV-1_ADA_ and on CXCR4 tropic HIV-1_MN_. Complement opsinization of both HIV-1_ADA_ and HIV-1_MN_ increased the binding and uptake by IDC (**Fig. 1C**) and MDC (data not shown). In subsequent experiments we investigated both the binding (interaction with cell surface receptors) and uptake into the DC (uptake via endocytic events). When binding and uptake of C-HIV and C-IgG-HIV was evaluated against the levels for F-HIV, they showed significantly increased binding to IDC (C-HIV 60% p<0.0005, and C-IgG-HIV 89% p<0.005) and MDC (C-HIV 28% p<0.0005, and C-IgG-HIV 50% p<0.05), whereas antibody opsonization of the virus had no or slight enhancing effect on the binding and uptake (**Fig. 1D**). The augmentation of the attachment and/or internalization by complement opsonization and complement in combination with anti-HIV-1 antibodies was stronger in IDC compared to MDC (**Fig. 1D**). We detected a similar binding and uptake pattern after extended incubation (24 h) with an increased uptake of C-HIV by both IDC (55% p<0.005) and MDC (64% p<0.05) (**Fig. 1E**). Only 39% of the free virions and 46% of C-HIV were localized in compartments protected from trypsin-EDTA treatment at 2 h in IDC (**Fig. 1F**), whereas more than 80% of the free virions and 96% of the complement opsonized virions were protected in MDC at this time point (**Fig. 1F**). The virions redistributed in IDC and 79% of the F-HIV and 85% of the C-HIV virions were protected against trypsin-EDTA treatment after 24 h incubation (**Fig. 1F**). For MDC over 95% of the virions were localized in compartments protected from trypsin-EDTA at 24 h (**Fig. 1F**). Assessment of the subcellular localization of the free vs. complement opsonized green fluorescent protein (GFP)-HIV-1 in IDC and MDC by confocal microscopy after 2 h incubation revealed strikingly different distribution of virions in IDC vs. MDC. As shown previously [14], [15], immature cells had many small vesicles containing HIV-1, whereas MDC had one or a few larger compartments containing HIV-1 viruses (**Fig. 1G**). There were no differences seen between F-HIV and C-HIV treated cells regarding the localization pattern in IDC or MDC (**Fig. 1G**). The treatment with trypsin-EDTA removed all surface located and trypsin sensitive virions on IDC and MDC and clearly showed that MDC had sequestered a greater amount of virions into trypsin insensitive compartments and that different mechanisms were involved in the binding and uptake of HIV-1 in IDC and MDC [5]. After 2 h incubation (**Fig. 1G**) HIV was located in compartments that should correspond to the ones described by Felts et al and Yu et al that still have continuity with the extracellular environment [15] [16].

### CD4 is a receptor for HIV-1 binding and internalization into IDC and MDC

**Figure 2:**
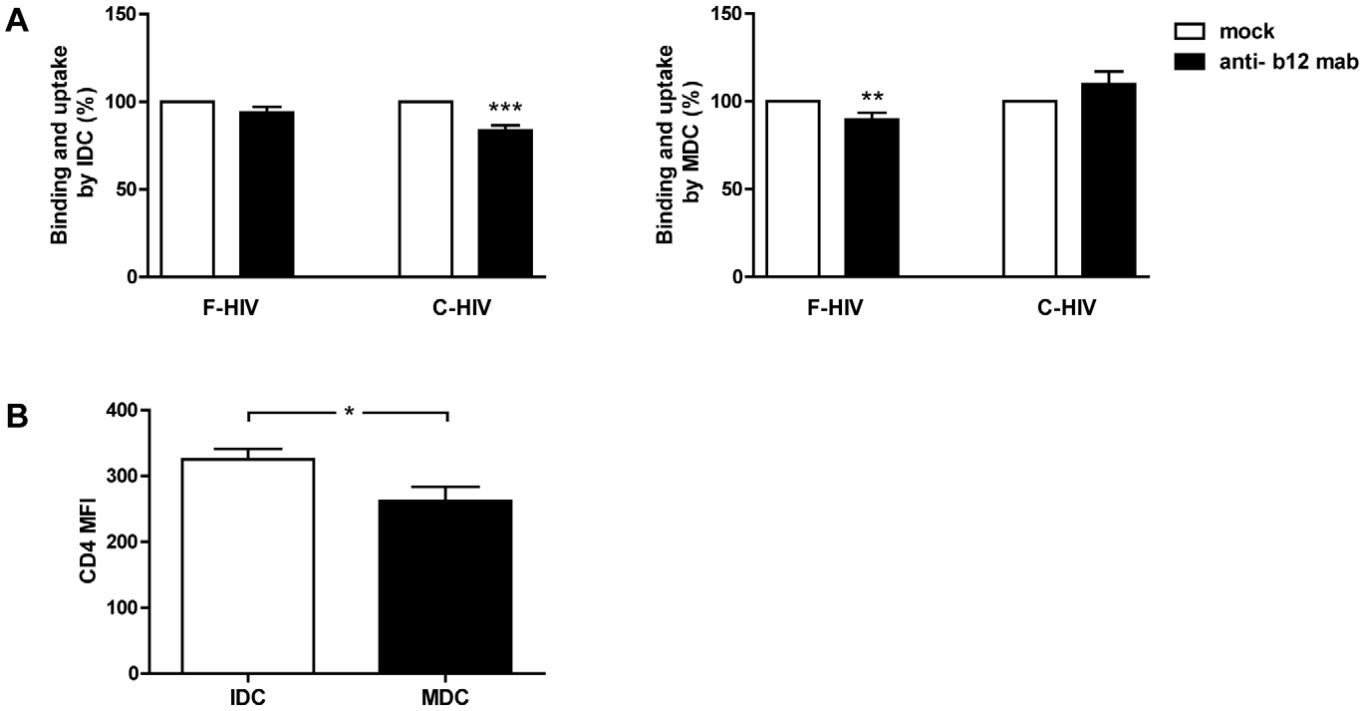
CD4 was utilized differently by free and complement opsonized HIV-1. (**A**) 0.15×10^6^ IDC and MDC were incubated with 50 ng p24I free HIV-1_BaL_ (F-HIV) and complement opsonized HIV-1_BaL_ (C-HIV) that had been pretreated with mock or b12 mAb (20 ug/ml) and the levels of bound and internalized HIV-1 was measured after 2 h by p24 ELISA (N = 15–17). (**B**) IDC and MDC were stained with fluorochrome conjugated anti-CD4 mAb and the surface expression of CD4 was measured as mean fluorescence intensity (MFI) by flow cytometry (N = 3). Results were tested for statistical significance using a two-sided paired t-test and p<0.05 was considered statistically significant. All values ± SEM. * = p<0.05, ** = p<0.005, *** = p<0.0005.

Multiple receptors and mechanisms are involved in the binding and uptake of HIV-1 and infection by C-HIV can be CD4 independent [17]. Of note, CD4 has been shown to be a major receptor for myeloid and plasmacytoid DC binding of HIV-1 [18], whereas it is less involved for binding to monocyte derived DC [18]. To evaluate the involvement of gp120-CD4 interactions in the binding and uptake of F-HIV and C-HIV by monocyte derived DC, virions were incubated with neutralizing anti-gp120 mAb b12 (20 ug/ml). Our data implicate CD4 in binding and internalization of C-HIV by IDC as a modest but significant (16% p<0.0005) decrease was seen with b12 blockade, but no effect was observed for F-HIV (**Fig. 2A**). The same effects were seen when different concentrations of the mAb b12 (5–40 ug/ml) were used. In contrast, for MDC b12 blocking of virus pointed to involvement of CD4 in binding and uptake of F-HIV as b12 treatment decreased binding and uptake of F-HIV but not C-HIV by a modest but significant amount (10% p<0.005). In fact, the neutralization of HIV with b12 seemed to slightly but not significantly increase the binding and uptake of C-HIV by MDC (**Fig. 2A**). IgG opsonization of HIV did not affect the amount of virions bound and taken up by IDC or MDC (**Fig. 1B**), therefore it is unlikely that the effects caused by b12 on binding and uptake are due to interaction with the Fc-receptors themselves. The apparent difference between IDC and MDC for CD4 involvement in F-HIV and C-HIV binding and uptake cannot easily be explained by the slightly higher CD4 levels expressed by IDC compared to MDC (**Fig. 2B**), but may be related to the differences seen in internalization and uptake pattern between these cells.

### Integrins are involved in binding and uptake of free and complement opsonized HIV-1

**Figure 3:**
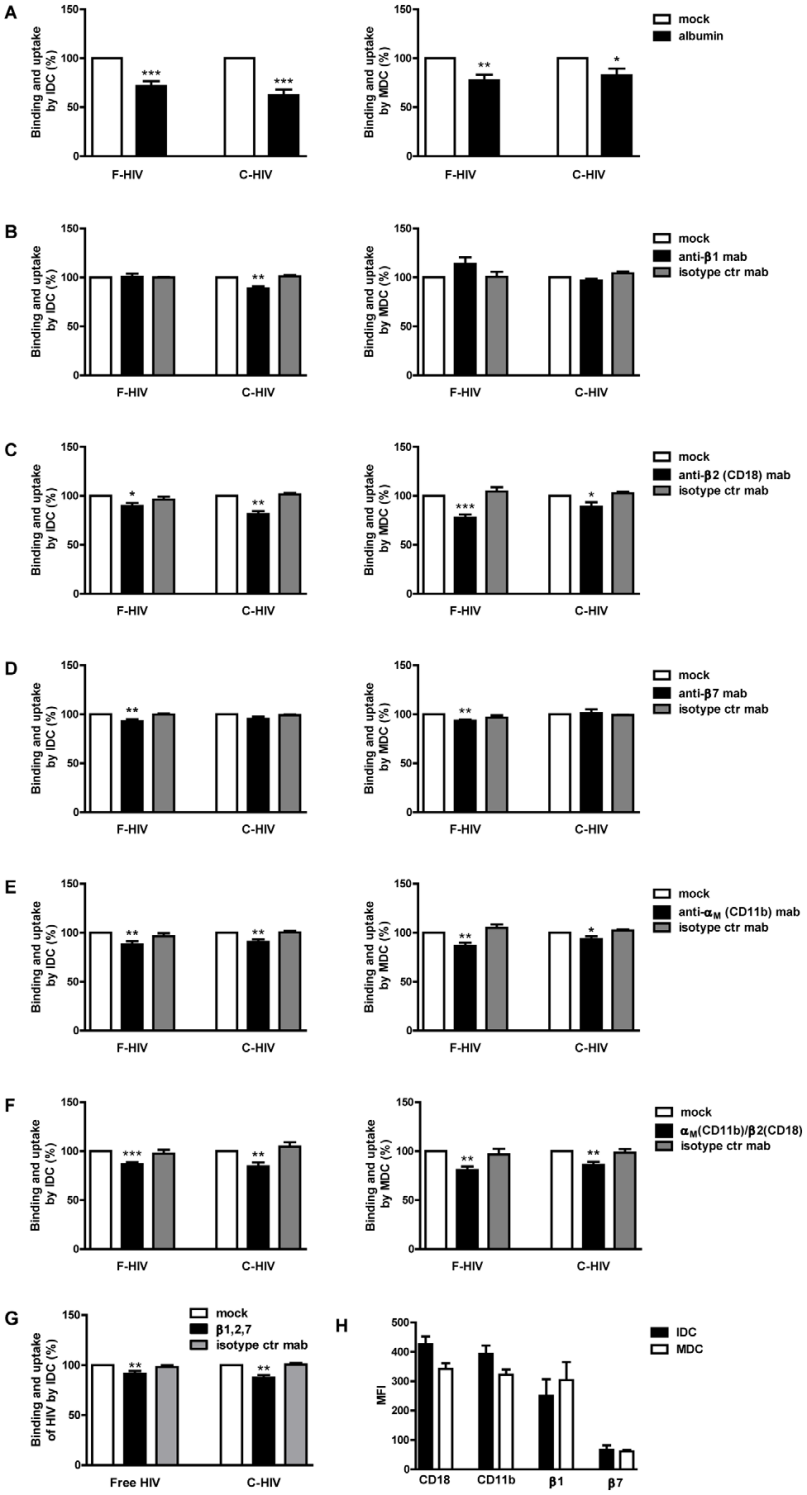
Integrins were used for binding and uptake of free and complement opsonized of HIV-1 by DC. (**A**) 0.15×10^6^ IDC and MDC were pre-incubated with albumin (2.5 mg/ml) or mock treated followed by incubation with 50 ng p24 free HIV-1_BaL_ (F-HIV) or complement opsonized HIV-1_BaL_ (C-HIV) and the level of binding and uptake of HIV-1 after 2 h was measured by p24 ELISA (N = 14–18). (**B–E**) IDC and MDC were pre-incubated with isotype control mAb or anti-β1 integrin mAb (**B**), anti-β2 integrin mAb (**C**), anti-β7 integrin mAb (**D**), or anti-α_M_ mAb (**E**) followed by incubation with F-HIV or C-HIV and the level of binding and uptake of HIV-1 after 2 h was measured by p24 ELISA (N = 4–10). (**F**) IDC and MDC were pre-incubated with isotype control mAb or mix of anti- α_M_, and β2 integrin mAbs followed by incubation with F-HIV or C-HIV and the level of binding and uptake of HIV-1 after 2 h was measured by p24 ELISA (N = 9–11). (**G**) IDC were pre-incubated with isotype control mAb or mix of anti-β1, β2, and β7 integrin mAbs followed by incubation with F-HIV or C-HIV and the level of binding and uptake of HIV-1 after 2 h was measured by p24 ELISA (N = 4). (**H**) IDC and MDC were stained with fluorochrome conjugated anti-α_M_ (CD11b) or anti- β2 (CD18) mAb and the surface expression was measured as mean fluorescence intensity (MFI) by flow cytometry (N = 3). All mAb were used at 20 ug/ml. Results were tested for statistical significance using a two-sided paired t-test and p<0.05 was considered statistically significant. All values ± SEM. * = p<0.05, ** = p<0.005, *** = p<0.0005.

Immune cells such as DC, B cells, and macrophages express different α- and β-integrins and several of these cell surface expressed molecules have been implicated in enhancing infection [4], [19] or cell to cell spread of HIV-1 [20]. Some of the integrins serve as building blocks for CR3 (CD11b/CD18), and CR4 (CD11c/CD18), while others are important receptors for cell attachment, e.g. VLA-4 (CD49d/CD29), or tissue specific homing, e.g. α4β7 [21]. We investigated the potential involvement of integrins in the binding and uptake of F-HIV and C-HIV by IDC and MDC. Blocking integrins with heat inactivated human albumin, which has been shown by Hidalgo et al to specifically block these receptors [22], significantly decreased the binding of F-HIV (28% p<0.0005) and C-HIV (38% p<0.0005) to IDC and MDC, (F-HIV (20%) p<0.005 and C-HIV (18% p<0.05)) (**Fig. 3A**). To determine the specific integrins involved in the binding of HIV-1 we investigated them individually using blocking antibodies. For IDC, we found an effect on F-HIV binding and uptake for β2- and β7-integrins (10%, p<0.05 and 7%, p<0.005, respectively) while β1- and β2-integrins were involved for C-HIV (11% and 9% p<0.005, respectively) (**Fig. 3B–D**). For MDC, blockade of β2- and β7-integrins significantly decreased F-HIV binding and uptake (22% p<0.0005 and 7% p<0.005, respectively), while C-HIV only interacted significantly with β2-integrins on MDC (11%, p<0.05) (**Fig. 3C–D**).

Blockade of the α_M_-chain of CR3 modestly but significantly decreased the binding and uptake of F-HIV and C-HIV by both IDC and MDC (**Fig. 3E**). We further evaluated the involvement of the complete CR3 (α_M_-integrin: CD11b and β2-integrin: CD18) in HIV-1 binding and uptake by MDC and IDC. It has previously been shown that the binding site for iC3b is located on the α-chain (CD11b) of CR3 [2]. Simultaneous blocking of α_M_-and β2-integrins inhibited C-HIV binding to IDC and MDC (16% p<0.005 and 14% p<0.005, respectively) (**Fig. 3F**) with similar results seen for F-HIV (13% p<0.0005 and 19% p<0.005, respectively) (**Fig. 3F**). These findings implicated involvement of both chains of CR3 in the binding and uptake of both F-HIV and C-HIV by DC (**Fig. 3C, E, and F**). Simultaneous blocking of β1-, β2-, and β7-integrins with antibodies gave a decreased binding and uptake for F-HIV (20% p<0.005) and C-HIV (23% p<0.005) (**Fig. 3G**). The inhibition of F-HIV and C-HIV binding to the individual α- and β-integrins on DC summed up to the blocking achieved when using heat inactivated albumin. No correlation was seen between the amount of bound virus and the mean fluorescence intensity (MFI) expression of CD11b and CD18 on IDC and MDC (**Fig. 3H**); the lower levels of CR3 expression on MDC compared to IDC did not seem to affect HIV-1 binding.

### Free and complement opsonized HIV-1 interacted with C-type lectins

**Figure 4:**
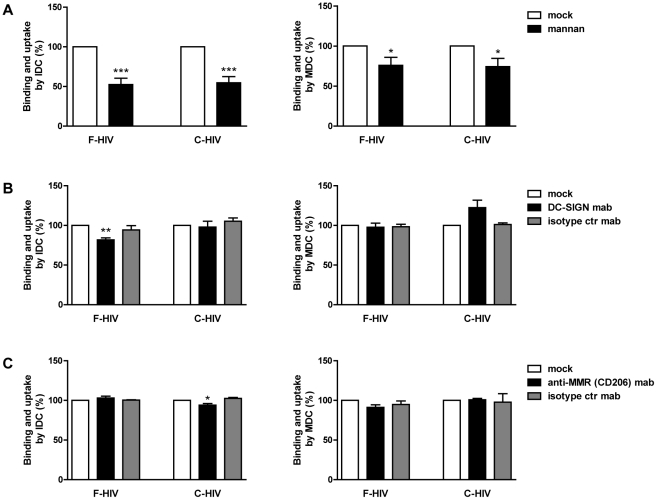
Both free and complement opsonized HIV utilized C-type lectins for binding and uptake by DC. (**A**) 0.15×10^6^ IDC and MDC were pre-incubated with mock or the C-type ligand mannan (10 mg/ml) followed by incubation with 50 ng p24 free HIV-1_BaL_ (F-HIV) or complement opsonized HIV-1_BaL_(C-HIV) and the level of binding and uptake of HIV-1 after 2 h was measured by p24 ELISA (N = 14–15). (**B–C**) IDC and MDC were pre-incubated with isotype control mAb or anti-DC-SIGN mAb (**B**), or anti-MMR mAb (**C**) followed by incubation with F-HIV or C-HIV and the level of binding and uptake of HIV-1 after 2 h was measured by p24 ELISA (N = 4–6). All mAbs were used at 20 ug/ml. Results were tested for statistical significance using a two-sided paired t-test and p<0.05 was considered statistically significant. All values ± SEM. * = p<0.05, ** = p<0.005, *** = p<0.0005.

The C-type lectin DC-SIGN has been implicated in the binding and uptake of F-HIV and C-HIV by DC and also in efficient infection and transfer of virus to T cells [9], [23]. Of note, the transfer of C-HIV also involved CR3 [9]. Besides DC-SIGN, other C-type lectins such as DEC-205, MMR, and DCIR have been shown to be involved in the binding, uptake, and/or infection of DC [5], [24]. We investigated whether the binding and uptake of F-HIV and C-HIV involved C-type lectins using the ligand mannan, which competes for binding to C type lectins. Mannan significantly decreased binding and uptake by IDC for both F-HIV and C-HIV (48% p<0.0005 and 46% p<0.0005, respectively) (**Fig. 4A**). Binding and uptake of F-HIV and C-HIV by MDC was also significantly decreased by mannan, although to a lesser extent (24% p<0.05 and 26% p<0.05, respectively) (**Fig. 4A**). Specific blocking of the C-type lectin DC-SIGN (using blocking mAbs 120507 and AZND1) modestly but significantly decreased the binding of F-HIV to IDC (18% p<0.005), whereas no significant effect was seen for C-HIV (**Fig. 4B**). Blocking DC-SIGN had no effect on the amount of F-HIV bound by MDC but surprisingly resulted in modestly but not significantly increased uptake of C-HIV. Moreover, blocking of MMR resulted in a small but significant decrease in binding of C-HIV by IDC (6% p<0.05), but no effect was seen for F-HIV (**Fig. 4C**). No involvement of MMR was seen for binding of F-HIV or C-HIV to MDC (**Fig. 4C**). These findings indicate that other C-type lectins than DC-SIGN and MMR are involved in the binding and uptake of F-HIV and C-HIV and this needs further elucidation.

### C-type lectins, integrins, and CD4 receptors in collaboration contributed to majority of HIV-1 binding and uptake by IDC and MDC

**Figure 5:**
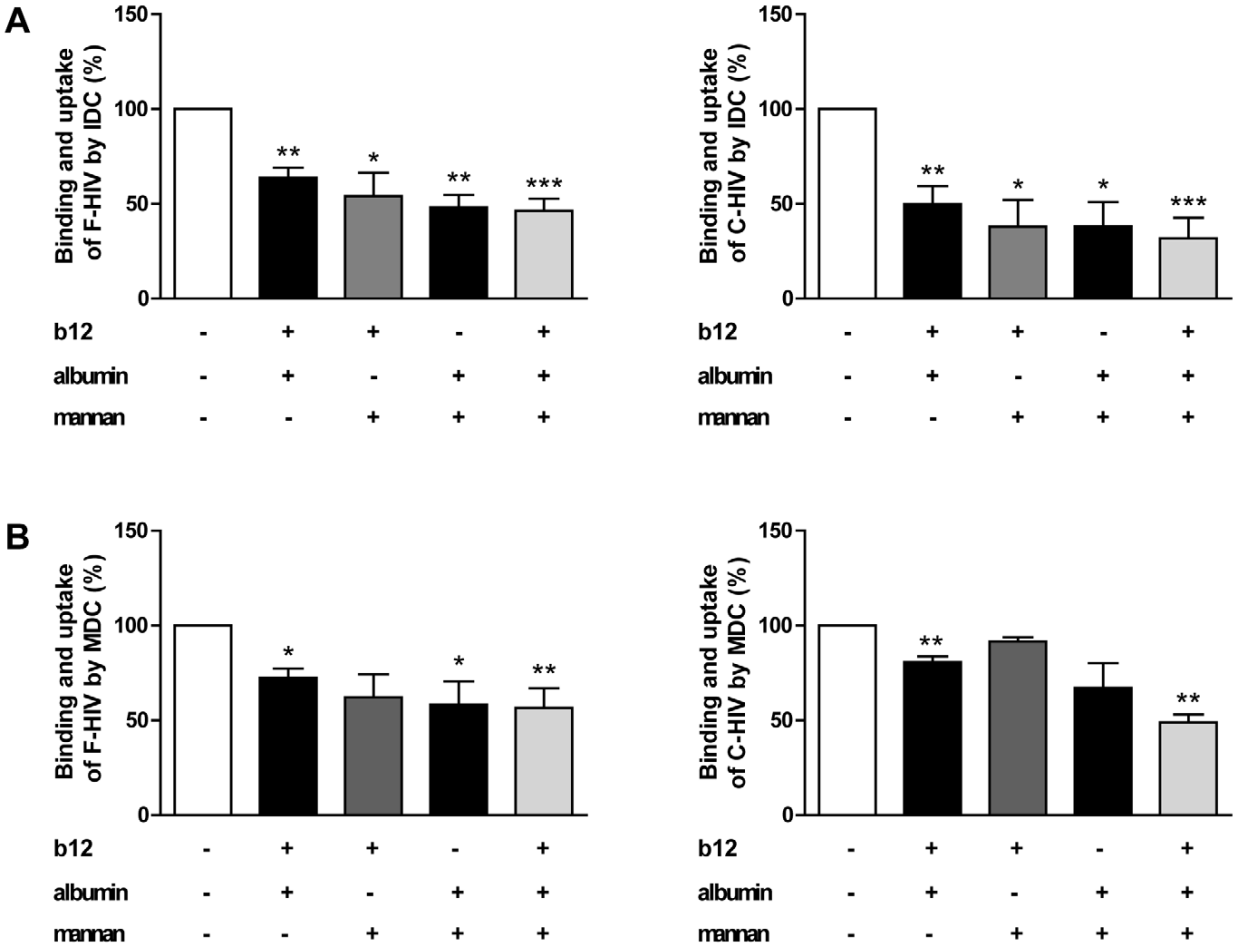
Blocking CD4, integrins, and lectins simultaneously significantly decreased DC binding and uptake of both free and complement opsonized HIV-1. 0.15×10^6^ IDC (**A**) and MDC (**B**) were pre-incubated with mock or various combinations of albumin (2,5 mg/ml) and/or mannan (10 mg/ml) and exposed to 50 ng p24 free HIV-1_BaL_ (F-HIV) or complement opsonized HIV-1_BaL_ (C-HIV), that had been mock or pretreated with b12 (20 µg/ml), and the level of binding and uptake of HIV-1 after 2 h was measured by p24 ELISA (N = 4–9). Values have been normalized and free HIV has been set to 100%. Results were tested for statistical significance using a two-sided paired t-test and p<0.05 was considered statistically significant. All values± SEM. N = 3–50. * = p<0.05, ** = p<0.005, *** = p<0.0005.

**Figure 6:**
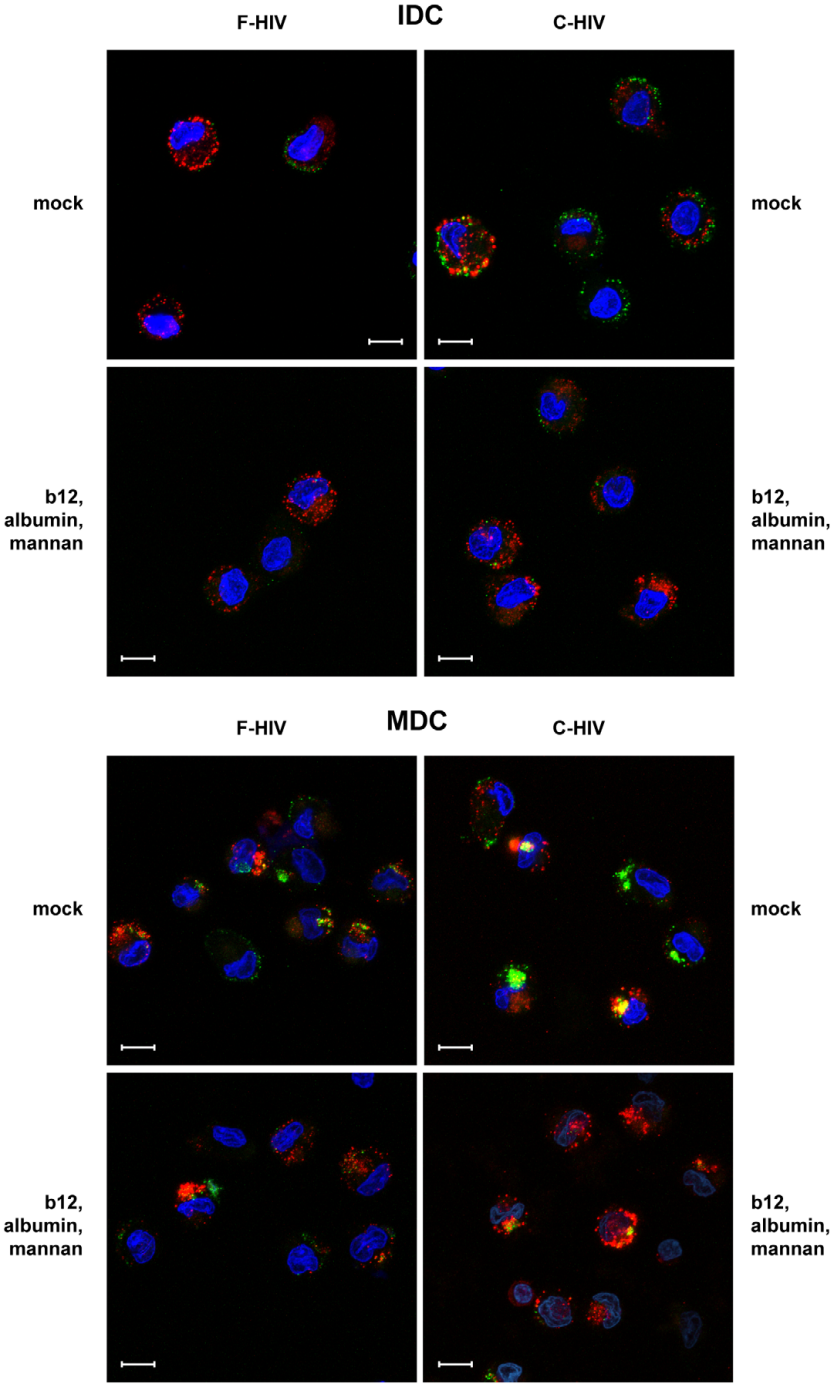
Blocking CD4, integrins, and lectins simultaneously visibly alters amount but not subcellular localization of free and complement opsonized HIV-1. 0.15×10^6^ IDC and MDC were pretreated with mock, b12 (20 µg/ml), albumin (2.5 mg/ml), and/or mannan and incubated with free or complement opsonized GFP-HIV-1_BaL_ (green) for 2 h, stained with mAb targeting the CD1a receptor (red) and DAPI (nucleus: blue). The binding and uptake of GFP-HIV-1_BaL_ was assessed by confocal microscopy.

It is clear from our study and several others that HIV-1 is promiscuous when it comes to the receptors it can utilize for cellular attachment and uptake by DC (**Fig. 2** **,** **3** **,** **4**) [4], [5]. Therefore, we investigated whether concurrent blocking of C-type lectins, integrins, and CD4 would exceed the effects seen for individual blockades on the binding of F-HIV and C-HIV to DC. For IDC the combination of mannan, and albumin, which blocked binding and uptake of F-HIV by more than 52% (p<0.005) (**Fig. 5A**). Binding and entry of F-HIV seemed to be CD4-independent as blockade of CD4/gp120 interactions did not decrease binding beyond the decrease mediated by blockade of only integrins and C-type lectins, which fits the findings for blockade of only CD4 binding with b12 (**Fig. 2A**). In IDC the combination of mannan, albumin, and b12 was more effective than the two blocker combination and gave the greatest decrease in the binding and uptake of C-HIV (68% p<0.0005) and the combination of mannan and albumin, or mannan and b12 gave similar decrease (∼62%), whereas the combination of albumin and b12 was less efficient with only 50% inhibition (**Fig. 5A**). These findings point to involvement of all three receptor groups, i.e. CD4, lectins, and integrins in the DC binding of C-HIV. In MDC binding and uptake of F-HIV was significantly blocked using the combined blockers b12 and albumin, albumin and mannan, and b12, albumin, and mannan (**Fig. 5B**). For C-HIV the highest impact was when the combined blockers albumin and mannan and b12 was utilized (**Fig. 5B**). To visualize the effects on uptake and subcellular localization of these blockers in IDC and MDC, GFP labeled F-HIV and C-HIV were used. The effects of b12, albumin, and mannan blockade on binding, uptake, and subcellular localization were assessed by confocal microscopy (**Fig. 6**) and showed a clear decrease in the amount of virions in DC, whereas no difference in localization could be detected (**Fig. 6**).

### Complement opsonized HIV-1 was more efficiently internalized by IDC and MDC compared to free virus despite similar amount of surface bound virions

**Figure 7:**
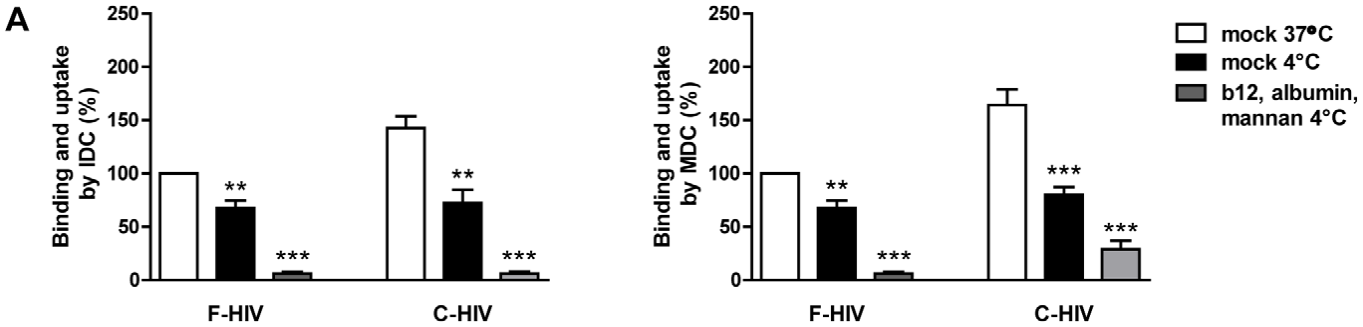
Complement opsonized HIV-1 was more efficiently internalized by DC. 0.15×10^6^ IDC and MDC were pretreated with mock or a combination of blockers albumin (2.5 mg/ml), and mannan (10 mg/ml). 50 ng p24 free HIV-1_BaL_ (F-HIV) or complement opsonized HIV-1_BaL_ (C-HIV), that had been mock or pretreated with b12 (20 µg/ml), was added to the different samples and incubated for 2 h in 37°C or 4°C before measuring the binding and uptake using a p24 ELISA (N = 6). Values have been normalized and free HIV has been set to 100%. Results were tested for statistical significance using a two-sided paired t-test and p<0.05 was considered statistically significant. All values ± SEM. * = p<0.05, ** = p<0.005, *** = p<0.0005.

We found that the binding and internalization of C-HIV was significantly higher compared to F-HIV and wanted to study the amount of virions that could bind to the DC cell surface at 4°C., since the internalization machinery is inactive at this temperature. Binding of virions to the IDC surface at 4°C was similar for F-HIV and C-HIV (68% and 72%, respectively) and for MDC (68% and 80%, respectively) (**Fig. 7**), which indicated that the increased levels of C-HIV bound and internalized by IDC and MDC were due to enhanced uptake process for C-HIV. Since our experiments indicated that both F-HIV and C-HIV used CD4, C-type lectins, and integrins for binding and uptake we blocked these receptors and assessed their role at 4°C. This abolished nearly all binding and uptake by DC indicating that these receptor families account for all or almost all F-HIV and C-HIV binding to DC (**Fig. 7**).

## Discussion

We showed that although F-HIV and C-HIV-1 bound to DC in comparable amounts at 4°C and utilized a similar receptor repertoire, internalization into DC was significantly enhanced when the virions were opsonized with complement fragments alone or in combination with HIV-1 specific antibodies. Blocking CD4 together with C-type lectins, and integrins abolished nearly all binding of HIV-1 to the cells, which indicated that we have identified all or almost all the receptor families involved in binding of F-HIV and C-HIV to DC. However, opsonization of HIV-1 did affect the repertoire of receptors used within the integrin and C-type lectin receptor families. DC maturation status had a large impact on both the capacity to bind and to internalize virus, with MDC binding higher amounts of virions and internalizing them more rapidly than IDC. In IDC, the majority of the virions remained in trypsin sensitive compartments [16] and/or at the cell surface after a short exposure, whereas they were redistributed into trypsin insensitive cellular compartments after a prolonged incubation. In contrast, in MDC the majority of the virions were present in trypsin insensitive compartments already within 2 h.

The potential relevance of opsonization of virions in vivo, both at sites for initial infection and during ongoing infection, is supported by the presence of complement components in mucosal secretions from cervix, and rectum and seminal fluid. Complement opsonization affects the binding pattern of both gp120 and host cell derived surface molecules incorporated into the viral envelope, thereby potentially affecting the infectivity of the virus [2]. Our data point to increased uptake and infection (data not shown) as both opsonization with complement alone or with antibody and complement enhanced the amount of virions taken up by IDC and MDC. One possible reason behind the enhanced uptake of opsonized HIV-1 could be the interaction between iC3b deposited on the viral surface and CR3 (CD11b/CD18) or other complement receptors on the DC, as iC3b on particle surfaces has previously been shown to enhance phagocytosis [25], [26]. The exact mechanisms behind iC3b mediated uptake have not yet been fully elucidated but may involve macropinocytosis [27], or phagocytosis as a result of the activation of CR3 (reviewed by [28]). The enhanced uptake of complement opsonized virions might be one explanation for more efficient transfer of infectious virions from DC to T cells [9]. Furthermore, the fact that iC3b can reduce inflammation and even promote tolerance [25], [29], implicates that the effect of complement opsonization on the HIV-DC interaction may have important consequences for the immune response against the virus.

The mechanisms for the enhancement of in vitro infection reported for C-HIV [2], [7], [9], [11] may involve focusing the virions via complement to cholesterol rich membrane areas with increased densities of CD4 and coreceptor to facilitate infection both at the cell surface and possibly in the endosomal compartment [4], [30], [31]. Moreover, the increased infection seen for C-HIV could also be due to enhanced levels of C-HIV taken up by MDC or decreased viral proteolysis (data not shown), with increased persistence of infectious virions. Blockade of the CD4 binding site on HIV-1 decreased the ability of C-HIV but not F-HIV to bind to IDC, but decreased binding of F-HIV and not C-HIV to MDC, findings that are not easily explained but that could depend on the differences seen in internalization and uptake pattern between these cells. Of note, complement opsonization can sterically hinder sites on the gp120 protein such as the V3 and V1/V2 regions [32] and this may influence binding via CD4, lectins, and integrins. Fc receptors can also enhance the binding and uptake of IgG opsonized HIV-1 by IDC as shown by van Montfort et al [33]. However, we did not see this effect using IgG-opsonized HIV-1 and therefore we find it unlikely that the result by b12 neutralization of HIV-1 is caused by the antibody itself but rather the effect of blocking the CD4 binding site on gp120.

However, as we showed, other receptors, besides the CD4 receptor, are also capable of gp120 dependent uptake of HIV, i.e. C-type lectins, such as DC-SIGN, and MMR [18], and even integrins [20].

Several receptors have been shown to be capable of enhancing the infectivity of F-HIV under various conditions, including LFA-1 [19], αV-integrin [34], and for C-HIV, CR3 [2], [8], [9], [17]. The α4β7-integrins, expressed on both DC and T cells, can interact with HIV-1 on T cells inducing cell signaling through this receptor resulting in enhanced HIV cell-cell spread [20]. Of note, α4β7-integrins are located on immune cells trafficking to the initial sites of infection [35]. This further implicates interactions between HIV-1 and integrins in biologically important communication with immune cells. Several of the integrin ligands, e.g. complement fragments, heparin, fibronectin, and fibrinogen [36], can be found in different body fluids and may influence viral infections by either coating the virions or the target cells. We found evidence for involvement of different integrins in DC binding and uptake of both F-HIV and C-HIV, with β1-integrin and β2-integrin involved in uptake of C-HIV and β1-integrin and β7-integrin involvement for F-HIV. Using albumin to block integrins resulted in a decrease similar to the sum of the effects seen using individual blockers against β1-, β2-, and β7-integrins, suggesting that these integrins are the main integrins involved in interactions with virions. To our knowledge, this is the first time HIV-1 has been shown to interact with β7-integrin on DC. In addition, our data prove that it is not only the α_M_-integrin chain (CD11b) of CR3 that is involved in the binding of C-HIV; in some settings the β-integrin (CD18) seems to play an even bigger role. We can't rule out that the CD18 involvement was due to binding to CR4 and/or LFA-1 and not to CR3. The CR3 involvement for F-HIV may depend on the interaction between host derived proteins in the viral envelope rather than HIV-1 gp120.

The C-type lectins have been shown to be important in the DC spread of the virus to T cells [23], [24]. Our findings show that mannan and anti-DC-SIGN mAbs did not affect the attachment of C-HIV to IDC and this concurs with a previous study by Pruenster et al [11] but is at odds with the findings by Bouhlal et al [9]. The decrease in binding and uptake when blocking DC-SIGN and MMR was considerably smaller than the decrease achieved when blocking with the lectin competing ligand mannan, suggesting that other C-type lectins may also be involved in the binding and uptake of HIV, such as syndecan 3, or DCIR.

HIV-1 attachment to host cells can involve multiple independent pathways and receptor groups [5], [37] and given this redundancy, blockade of a single receptor group may not impact the amount of virions bound. Simultaneous inhibition of several receptor groups decreased the F-HIV and C-HIV binding and uptake by IDC and MDC. The mechanisms for the entry of HIV-1 into MDC need to be further elucidated, as at 37°C neither the single nor combined blocking of receptors could affect F-HIV or C-HIV binding to a greater extent in our system, which may suggest that different mechanisms are utilized for HIV-1 internalization in MDC compared to IDC.

Recent studies have demonstrated that IDC have a more rapid degradation of virions and reduced trans-infection ability compared to MDC [9]. Complement opsonization has been shown to enhance HIV trans-infection by MDC [9], suggesting a that longer preservation time inside the cell may give rise to an increased trans-infection. The discrepancy in internalization and degradation seen for free and C-HIV might be due to the differences in their receptor usage and efficiency in uptake, which may lead to different pathways and degradation routes inside the cell. However, other mechanisms, e.g. macropinocytosis, might also affect the uptake of F-HIV and C-HIV leading to a more rapid degradation process of free virions.

Furthermore, the increased uptake of complement and immune complexed HIV-1 may also lead to enhanced MHC I and II presentation. The opsonization of the virions may affect the MHC I and II antigen presentation by guiding the HIV to different paths inside the DC and some evidence exists for changed MHC II presentation where the co localization with HLA DR changes with a decrease for complement and enhancement for antibody and complement antibody opsonized HIV-1 [2], [10]. The effect of HIV-1 opsonization on antigen presentation is now under investigation.

In summary, the increased uptake, but also the prolonged degradation time, will have an influence on the cellular transportation of complement opsonized virus to the lymph nodes, infection of the host, trans infection, and antigen presentation by DC. Taken together all these different factors will influence the HIV-1 pathogenesis and must be taken into consideration when developing HIV-1 drugs and vaccines.

## Materials and Methods

### Culture medium, cytokines, and reagents

Culture medium RPMI-1640 (Fisher scientific, Gothenburg, Sweden) was supplemented with 20 ug/ml gentamicin (Fisher scientific), 10 mM HEPES (Fisher scientific), and 1% human plasma. Recombinant human GM-CSF (Genezyme) and recombinant human IL-4 (R&D Systems, Minneapolis, MN) were utilized for in vitro propagation of DCs.

### Propagation of monocyte derived DC

Leukocyte enriched Buffy coats were obtained from the department of clinical immunology and transfusion medicine, Karolinska University Hospital, Stockholm. PBMC were separated by density gradient centrifugation on Ficoll-Hypaque (Amersham Pharmacia Biotech, Piscataway, NJ). PBMC were incubated on tissue culture dishes for 1 h at 37°C to allow adherence of the DC progenitors. Non adherent cells were removed by washing the plates several times. The progenitors were differentiated into IDC by adding 100 IU/ml GM-CSF and 300 U/ml IL-4 at day 0, 2, and 4 of culture. Maturation was induced day 5 by adding 30 µg/ml poly-IC (Sigma Aldrich, Stockholm, Sweden) and culturing for an additional 48 h. On day 7, the DCs were assessed for expression of CD14 and CD83.

### Virus propagation

HIV-1_BaL_ (obtained from the NIH AIDS Research and Reference Reagent Program) was prepared from infected SUPT1 CCR5 Cl 30 cells (provided by Dr. Jim Hoxie, University of Pennsylvania) and purified and concentrated by sucrose gradient ultracentrifugation. Matching preparations of non-infectious HIV-1_BaL_ virions (Lots P4122 and P4243, Biological Products Core/AIDS and Cancer Virus Program, SAIC-Frederick, Inc., NCI Frederick,) were prepared by chemical inactivation with 2,2′-dithiodipyridine (Aldrithiol-2, AT-2); AT-2 inactivation eliminates infectivity by covalent modification of internal virion proteins but preserves conformationally and functionally intact envelope glycoproteins on the virion surface (16, 17). GFP HIV-1_BaL_ was prepared as described previously [38].

### Opsonization of HIV-1

HIV-1 was opsonized with complement fragments (C-HIV), HIV-specific IgG (IgG-HIV), or a combination of both (C-IgG-HIV). C-HIV was obtained by incubation of HIV-1_BaL_ (30 µg/ml p24) with an equal volume of human serum (HS) derived from healthy volunteers containing 25% Veronal buffer (0.6 mM CaCl_2_ and 0.9 mM MgCl_2_ (VBS2+)) [17]. To obtain C-IgG-HIV, 0.20 µg/ml neutralizing HIV-specific IgG (HIVIGLOB, SMI, Stockholm Sweden) [39] and 20 µg/ml gamma globulin (Pharmacia, Stockholm, Sweden) was added besides the HS containing Veronal buffer, whereas IgG-HIV was obtained by adding the mixture of HIV neutralizing IgG and gamma globulin Free HIV was treated with medium alone. All groups were incubated for 1 h at 37°C.

### Phenotypic characterization of DC

DC were stained with fluorochrome conjugated mAb against CD83, CD14, CD4, CCR5, CD11b, CD18, and the corresponding isotype controls (BD PharMingen, SanDiego, CA), assessed by Flow cytometry (FACS Calibur) and analyzed by FLowJo (Treestar, Ashland, OR, USA).

### Virus capture assay

0.15×10^6^ DC at a concentration of 1×10^6^ cells/ml were pre incubated for 30 min at 37°C with different binding and uptake inhibitors. The following inhibitors were used at the indicated concentrations: 10 mg/ml mannan, 2.5 mg/ml denaturated human serum albumin (Sigma-Aldrich) [40], 5 mg/ml EDTA, 2.5 mg/ml EGTA (Fisher Scientific), blocking mAb against the CD4 binding site on gp120 (b12, a kind gift from Professor D. Burton), complement receptor 3: CD11b (ICRF44: Ancell Corp. Bayport, USA), CD11b (KIM75) and anti-β2 integrin (CD18: clone 6.5) (kind gifts from M. Robinson, Celltech, UCB group), anti-human DC-SIGN (CD209) (120507 from R&D, and AZND1 from Beckman Coulter), anti-CD206 (clone 15-2: Biosite), anti-β7 integrin (clone F1B504: Biosite) and anti-β1 integrin (CD29) clone JB1A (Millipore), and matching isotype controls were used. The viability of the cells was assessed by trypan blue exclusion to rule out toxicity from any of the drugs or Abs used. The Abs were used at 20 µg/ml. After preincubation, 50 ng (p24^CA^ equivalent) of free- or C-HIV was added to the samples consisting of 1.5×10^5^ cells/group. The cells were incubated for 2 h at 37°C or 4°C and thereafter washed 2 times in RPMI-1640, trypsin or mock treated for 10 min at 37°C, an then washed an additional 3 times to remove unbound virus. The cells were lysed in 0.5% Triton X-100 and the amount of HIV-1 was determined by a p24 capture ELISA. The experiments were repeated at least four times in duplicates. For comparison of results obtained in different experiments, using cells from different donors, the binding and uptake of HIV-1 in DC in absence of inhibitors was normalized to 100% for each experiment.

### Immunofluorescence and confocal microscopy

IDC and MDC were incubated with GFP HIV_Bal_ and different blockers as described above (viral capture assay). The cells were stained at 4°C for 30 min with anti-human CD1a (HI149 from BioLegend, San Diego, CA) or anti-human HLA-DR (Beckman Coulter) mAb. The stained cells were washed 3 times and stained with a secondary Ab (Rhodamine Red-X conjugated donkey anti-mouse IgG, Jackson ImmunoResearch, Baltimore, PA) for 1 h at RT. The samples were fixed in 4% paraformaldehyde for 10 min, washed, resuspended in PBS, transferred onto microscope slides and mounted using mounting medium for fluorescence with DAPI (Vector Laboratories, Burlingame, CA). The samples were analyzed by a LSM 510 META confocal microscope (Carl Zeiss AB, Stockholm, Sweden) using the LSM 510 software.

### Statistical analysis

GraphPad Prism 5 (GraphPad Software, La Jolla, CA) was used for statistical analysis of all samples. Results were tested for statistical significance using a two-sided paired t-test and p<0.05 was considered statistically significant. In all figures, N denotes the number of times each experiment was repeated, using cells derived from different healthy blood donors.

## Supporting Information

Figure S1 **50 ng p24 HIV-1_BaL_ was incubated with mock, fresh human sera or heat-inactivated human sera and added to 0.15×10^6^ IDC and MDC.** The level of binding and uptake of HIV-1 after 2 h was measured by p24 ELISA. Results were tested for statistical significance using a two-sided paired t-test and p<0.05 was considered statistically significant. Values have been normalized and free HIV has been set to 100%. All values± SEM. * = p<0.05, ** = p<0.005, *** = p<0.0005.
(TIF)
