# Supplementary figures and images for: Complement Opsonization of HIV-1 Enhances the Uptake by Dendritic Cells and Involves the Endocytic Lectin and Integrin Receptor Families
Source: PLoS One. 2011 Aug 11;6(8):e23542. doi: 10.1371/journal.pone.0023542 (PMC3154940; doi:10.1371/journal.pone.0023542)

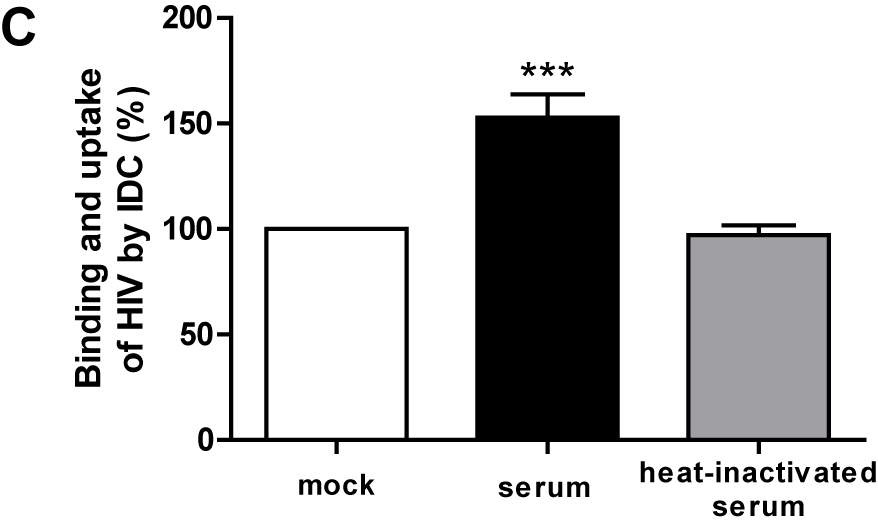

Supplement: Figure S1 — 50 ng p24 HIV-1BaL was incubated with mock, fresh human sera or heat-inactivated human sera and added to 0.15×10^6^ IDC and MDC. The level of binding and uptake of HIV-1 after 2 h was measured by p24 ELISA. Results were tested for statistical significance using a two-sided paired t-test and p<0.05 was considered statistically significant. Values have been normalized and free HIV has been set to 100%. All values± SEM. * = p<0.05, ** = p<0.005, *** = p<0.0005. (TIF) [file pone.0023542.s001.tif]
